# Nanoparticle Impact on the Bacterial Adaptation: Focus on Nano-Titania

**DOI:** 10.3390/nano12203616

**Published:** 2022-10-15

**Authors:** Maria Grazia Ammendolia, Barbara De Berardis

**Affiliations:** National Center for Innovative Technologies in Public Health, Istituto Superiore di Sanità, 00161 Rome, Italy

**Keywords:** nano-titania, TiO_2_ NPs, bacterial adaptation, nanoparticle resistance, toxicity, risk assessment

## Abstract

Titanium dioxide nanoparticles (nano-titania/TiO_2_ NPs) are used in different fields and applications. However, the release of TiO_2_ NPs into the environment has raised concerns about their biosafety and biosecurity. In light of the evidence that TiO_2_ NPs could be used to counteract antibiotic resistance, they have been investigated for their antibacterial activity. Studies reported so far indicate a good performance of TiO_2_ NPs against bacteria, alone or in combination with antibiotics. However, bacteria are able to invoke multiple response mechanisms in an attempt to adapt to TiO_2_ NPs. Bacterial adaption arises from global changes in metabolic pathways via the modulation of regulatory networks and can be related to single-cell or multicellular communities. This review describes how the impact of TiO_2_ NPs on bacteria leads to several changes in microorganisms, mainly during long-term exposure, that can evolve towards adaptation and/or increased virulence. Strategies employed by bacteria to cope with TiO_2_ NPs suggest that their use as an antibacterial agent has still to be extensively investigated from the point of view of the risk of adaptation, to prevent the development of resistance. At the same time, possible effects on increased virulence following bacterial target modifications by TiO_2_ NPs on cells or tissues have to be considered.

## 1. Introduction

Nanomaterials are widely used in various industrial manufacturing processes and have applications in everyday consumer products. Their use is due to their small size and physicochemical properties leading to different properties in chemical and biological reactions by the enhancement of physical phenomena. In recent years, a great number of consumer products containing nanoparticles have reached the market, even though the impact on biological systems has still to be defined in regard to hazard and risk to public health.

Among metallic nanomaterials, Titanium dioxide (TiO_2_) nanoparticles (NPs) represent one of the most employed nanomaterials in several fields such as material science, environmental protection, agriculture, cosmetics, food, and medicine [1]. The use of nano-titania has been also proposed as an alternative antibacterial agent for antibiotic-resistant microorganisms [2] and several studies have been performed on the antibacterial properties of TiO_2_ NPs demonstrating that they have a good ability to kill bacteria alone or in combination with antimicrobials. However, these promising results are counterbalanced by data on emerging bacterial adaptation/resistance to these nanoparticles through different mechanisms of metabolic regulation [3].

**Figure 1:**
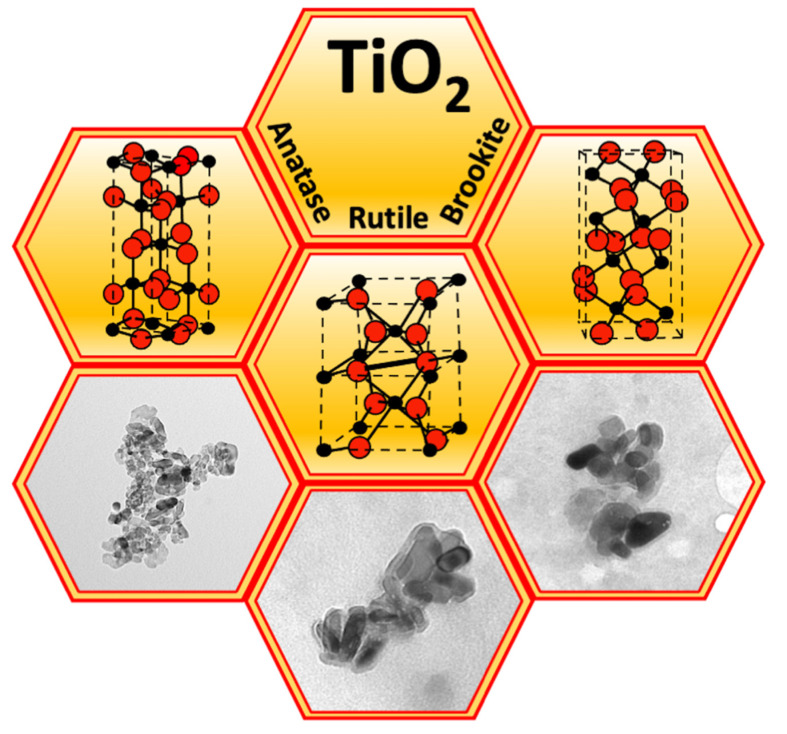
Crystalline structures of anatase, rutile, and brookite TiO_2_ (spheres: red O_2_, black Ti) and transmission electron microscopy images of the corresponding TiO_2_ NPs.

TiO_2_ is mainly present in three crystalline forms, rutile, anatase, and brookite, that exhibit different refractive index, stability, and photocatalytic activity (Figure 1). All three crystal structures are made up of distorted octahedra, composed of one Ti atom surrounded by six oxygen atoms. However, the arrangement of the octahedra is different in the three forms. In rutile each octahedron shares two opposite edges and the connecting angle, forming a tetragonal structure. In anatase, each octahedron shares four edges, giving rise to a more distorted tetragonal structure. In brookite, the octahedra share three edges and angles forming an orthorhombic structure [4]. Rutile is the predominant form of synthesised TiO_2_. It is stable and shows high dispersion and one of the highest refractive indices of any known crystal at visible wavelengths [5]. Anatase is the second form of synthesized TiO_2_ commonly used. It is metastable and when heated, it transforms into rutile [6]. Moreover, it has higher hydrophilicity and photocatalytic activity than rutile, probably due to the different distances between the Ti-O atoms of the two crystalline forms [7]. Brookite, like anatase, is metastable and changes to rutile when heated. It is the least synthesised form of TiO_2_, due to the difficulty to synthesize pure brookite without traces of anatase and rutile [6].

**Figure 2:**
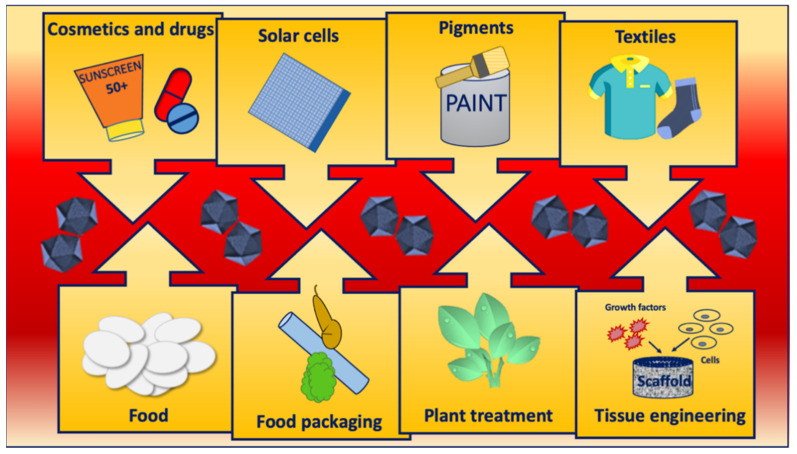
Main applications of TiO_2_ NPs.

Due to its interesting physicochemical properties, TiO_2_ is an excellent catalyst and, when activated by sunlight, is able to degrade numerous organic compounds by oxidation. TiO_2_ is also an excellent solar filter, able to absorb mainly the UV component of sunlight and to be transparent to visible light. In addition, it possesses fine mechanical properties, good corrosion resistance, and biocompatibility. For these characteristics TiO_2_ NPs are used in several products: in paints, plastics, paper, and rubber as a pigment; in self-cleaning and de-polluting surfaces, or the treatment of contaminated water as a chemical catalyst; in cosmetics as a solar filter; in drugs as an excipient; in food as a food additive and coloring agent; and in medical devices as a coating to prevent infection and inhibit biofilm formation, or as a ceramic material in scaffolds for bone tissue engineering, or in wound dressing (Figure 2). TiO_2_ NPs are embedded in membranes made up of natural and/or synthetic polymers with antimicrobial properties forming nanocomposites employed in wound healing. Polymers recently used in membranes incorporating TiO_2_ NPs include chitosan/poly(N-vinylpyrrolidone), salicylimine-chitosan, chitosan/pectin, and chitosan/poly(propylene glycol). All these biopolymers and synthetic polymer-based TiO_2_ nanocomposites have shown better performance than polymers without NPs, in terms of mechanical properties, biocompatibility, antimicrobial activity, and healing rate in wound dressing [8]. Other TiO_2_ NP applications include the textile sector to create novel functionalities by chemical modification of textile fibers, or for the photocatalytic decolorization of dyes in wastewater (Figure 2) [1,7].

The application of a specific TiO_2_ crystalline structure is related to its characteristics, such as density, refractive index, and photochemical reactivity. Due to its higher refractive index and density, rutile finds principal applications in pigments and cosmetics industries, whereas anatase is used in photocatalytic applications [4]. Recently, despite the difficulties in synthesising brookite, interest in this material has grown due to its promising photocatalytic properties in environmental remediation, hydrogen formation, Li-ion battery production, and organic synthesis [6].

Agriculture represents another field for the exploitation of TiO_2_ NP photocatalytic activity, to stimulate plant growth, induce greater stress tolerance in plants, promote the accumulation of mineral nutrients, trigger plant defences, or degrade pesticides. Moaveni et al. [9] found that the application of TiO_2_ NPs at very low concentrations, first during the stem elongation phase and then during the four-leaf phase of the barley plant, led to an increase in grain yield and weight. Recently, Ishad et al. [10] found that the application of TiO_2_ NPs is able to relieve the stress caused by cadmium in wheat grains, while Khattack et al. [11] showed the significant benefit of TiO_2_ NP use, combined with salicylic acid, on the growth of the sunflower plant under water stress conditions. Korosi et al. [12] demonstrated that TiO_2_ NPs, both in the anatase and in the rutile phase, at concentrations in the range 10–100 mg/L, are able to enhance the photosynthetic performance and the mineral nutrient level in grapevine leaves. They suggest their use in agricultural applications in low concentrations as potential elicitors. Amalraj et al. [13] showed the photocatalytic degradation of the two most popular and broadly used organophosphorus pesticides by TiO_2_ anatase NPs in the range of 9–26 nm under UV radiation.

### 1.1. TiO_2_ Nanoparticles in Cosmetics and Food

As particle size decreases, the specific surface area and the number of structural defects of nanoparticles increases, leading to higher chemical and biological reactivity of the material. TiO_2_ is today used only in its nanoform in sunscreen and some daycreams, foundations, and lip balms [14]. The nanoscale size of TiO_2_ brings some advantages in sun lotions and sprays, such as transparency to visible light and increased UV attenuation. Commercial forms of TiO_2_ show organic and inorganic surface modifications to facilitate dispersion in the matrices, enhance the resistance to photoactivity, and increase catalytic activity and UV absorption properties. In particular, both hydrophilic organic molecules such as glycerol, trimethylolpropane, and metal oxides, are used to improve dispersion, while treatment or coating with metal oxides reduces photoactivity in some plastics in order to prevent fading and degradation, or improve catalytic activity [5]. However, particle size, agglomeration, and surface treatment of TiO_2_ NPs affect their performance. Smaller, non-agglomerated, and particles coated to minimize surface effects, provide higher UV attenuation and stability in sunscreens [15].

TiO_2_ NPs are also widely used in many food categories. The Center for Food Safety identified 80 food products containing TiO_2_ NPs [16]. Both the anatase and rutile phases have been detected in E171, a food additive applied widely as a colorant in candies, sweets, and chewing gums, and also in pastries, low-fat dairy products, and sauces. Weir et al. [17] detected the highest concentrations of E171 in sweets and estimated that 17–36% of TiO_2_ particles detected in cookies and chewing gums were less than 100 nm in size. Similar results were obtained by Chen et al. [18] who detected the highest amounts of TiO_2_ in the sugar coating of the chewing gums. Transmission electron microscopy (TEM) analysis showed that TiO_2_ particles in gum ranged from 40 to 300 nm in size, with over 93% of the TiO_2_ particles <200 nm and about 40% < 100 nm. These observations are in agreement with another study, which showed a size distribution ranging from 20 nm to 400 nm [19]. Recent research, carried out by standardized and validated methods for the physicochemical characterization of E171, allowed the identification of significant fractions of nanoparticles in several E171 samples [20]. Moreover, 12 out of the 15 E171 materials studied were identified as nanomaterials according to the EC-recommended definition [21]. A study, performed using different analytical techniques, such as X-ray diffraction (XRD), Fourier Transform Raman spectroscopy (FT-Raman), Inductively Coupled Plasma Atomic Emission Spectroscopy (ICP-MS), and TEM, to determine TiO_2_ NP content in the coating of chewing gums, showed that the amount of TiO_2_ was dependent on the kind of product. In some cases, TiO_2_ constituted the entire coating, in others this metal oxide was embedded in organic matrices or mixed with minerals, such as talc, magnesium silicate, or calcium carbonate. TiO_2_ NPs represented about 19% of all TiO_2_ particles constituting the coatings, with a mean size of about 135 nm [22]. Although the European Food Safety Authority (EFSA) considered the TiO_2_ absorption by the gut intestinal tract as being very low, and authorized the use of E171 [23], in 2020, the French Food Safety Agency banned it in food products entering the country [24]. In May 2021, the EFSA revised the previous safety assessment of E171, following a request from the European Commission, and established that, based on new studies and stronger scientific methods, it can no longer be considered safe as a food additive. It is not possible to set a safety level for the daily intake of E171 since risks related to genotoxicity cannot be excluded [25].

Recently, a growing interest has been directed to the study of the changes in NP physicochemical properties due to food processing methods and interactions with food matrices. In fact, biological molecules in food matrices, such as proteins, carbohydrates, phospholipids, and lipids, interact with NPs and, depending on their affinity to the NP surface, can be adsorbed, leading to the formation of a biomolecular corona. The formation of the biomolecular corona is affected by the size, shape, charge, and hydrophobicity of NPs, the properties of the biomolecules, such as molecular weight, charge, hydrophobicity, and the pH, temperature, and ionic strength of the environment [26]. It has been shown that during the interaction with biological molecules, the NPs undergo changes in size, agglomeration, interfacial composition, and electrical charge that have a direct impact on their gastrointestinal fate, absorption, accumulation, and toxicity [24,27]. Bing et al. [28] showed that the changes in surface charge and size of TiO_2_ NPs, as well as the structure and thickness of protein corona, depended on the type of protein absorbed on the NP surface. The interactions of TiO_2_ NPs with gliadin improved the NP stability and led to the formation of large clumps containing embedded NPs, while the absorption of gluten decreased the zeta potential and resulted in the formation of thick homogeneous layers around the NPs. Literature data about the interaction between proteins adsorbed on the TiO_2_ NP surface and their changes in physicochemical characteristics showed altered behavior of ingested NPs in the human gastrointestinal tract. A study investigating the formation of protein corona due to the interaction of casein with TiO_2_ NPs highlighted changes in NP surface charge, aggregation status, and interfacial composition, and alterations of the nature of the peptide bonds exposed to digestive enzymes, with a consequential reduction in the rate of gastric protein digestion [29]. Moreover, the TiO_2_ NPs interfacial composition may change when they pass through the gastrointestinal tract due to protease activity or co-adsorption processes, such as that of digestive enzymes, affecting their activity [30,31]. Reductions in the activity of lipase and trypsin in the presence of TiO_2_ NPs have also been reported [27,32].

Regarding nano-titania biodistribution, it was observed that orally-administered TiO_2_ NPs penetrated the bloodstream and were distributed in different organs and tissues [33]. In vivo studies showed that E171 distributed rapidly from the systemic circulation to various tissues and mainly affected the liver and intestines. Bettini et al. [34], assessing the tissue distribution of food-grade E171, orally administered at 10 mg/kg body weight/day to rats over seven days, observed TiO_2_ NP accumulation in the liver and the transepithelial passage in the jejunum and colon. Talamini et al. [35] showed that repeated oral exposure for three weeks of E171 to mice for three days/week at 5 mg/kg body weight led to their significant accumulation in the liver and intestines. Finally, in a recent study, Han et al. [33], determining the biodistribution of E171 in the colon, kidney, and spleen harvested from rats exposed to 1000 mg/kg food-grade E171, administered daily by oral gavage for 90 days, observed TiO_2_ NP accumulation only in the colon, indicating this organ as the main excretion route. Although most ingested NPs are excreted via the feces, a certain number of the nanoparticles penetrate the cells of the gastrointestinal tract and can induce oxidative stress. Inflammatory response and liver disfunction caused by oxidative stress were observed by Cui et al. [36] after TiO_2_ NP 90-day oral exposure. Microinflammation and initiated preneoplastic lesions in the colon were also found after 100-day repeated dose exposure of E171, promoting altered expression of genes involved in innate and adaptive immune response and oxidative stress [37].

### 1.2. TiO_2_ NPs in the Environment

The large production of TiO_2_ NPs can lead to their increasing release into the environment. Release into the aquatic environment can occur from building facades through urban runoff [38], from sunscreen creams during recreational activities, such as swimming or bathing, as well as from personal care through wastewater, but also from food products containing the food additive E171, or textiles [39]. Additional release comes from essential nutrients for plant growth and development containing TiO_2_ NPs via agricultural runoff [40]. The behavior and transformations of TiO_2_ NPs once they enter the aquatic environment, depend not only on the chemical properties of the NPs, such as the coating added to ensure their stability, but also on factors related to the surface of the water, such as currents, waves, and salinity, that influence their bioavailability and impact on the aquatic environment [41]. Moreover, the NPs do not remain suspended for a long time in the water because they disperse, aggregate, and settle in the sediments, and interact with aquatic microorganisms and natural colloids.

Data on NP concentrations in different environmental compartments, largely based on modelling approaches [42,43,44], estimate very low concentrations in surface waters, ranging from ng to low μg/L. In the last 10 years, interest has grown in evaluating the release of NPs, especially TiO_2_ and ZnO, and approaches combining different analytical methods have been developed allowing the identification of their release from sunscreens. Data available in the literature on TiO_2_ NP levels are in agreement with predicted concentrations. A study conducted in waters near the coast of Majorca estimated a release in the surface microlayer of Ti from the sunscreens used by bathers at concentrations in the range 6.9–37.6 µg/L. The authors showed that the dissolution of sunscreens led to the release of other inorganic elements, like Si, N, and P, with a potential stimulation of algae growth [45]. Reed et al. [46], monitoring TiO_2_ NP concentrations in the Clear Creek River in Colorado, showed values in the range 0.4–110 ng/L, due to NP release from personal care products. Results from one-year observations indicated a concentration between 1.7 to 27.1 μg/L of TiO_2_ NPs in the waters of the Old Danube lake [47]. Loosli et al. [48], by use of an analytical approach combining single particle-inductively coupled plasma-mass spectrometry (SP-ICP-MS) and TEM, found concentrations of TiO_2_ NPs up to 100 μg/L in surface waters impacted by sanitary sewer overflows in the United States. Another study on the TiO_2_ NP level in river waters and sediments in sampling sites characterized by agricultural and industrial activities showed TiO_2_ NP mass concentrations of up to 7 µg/L in river water samples, and 871 µg/g in river sediment samples [40]. Finally, data from research conducted on the fate of anatase nanoparticles used to photodegrade the dyes from the wastewater of the textile industries, showed that 90% of the NPs were bioabsorbed on the activated sludge of the treatment plants, and then reached the environment via landfill [49].

## 2. TiO_2_ NP Antibacterial Activity

Nano-titania is promising in both the medical field and industry due to a reduction in bacteria growth and infections. By combining antibiotics and nano-titania it could be possible to decrease infections and bacterial growth in orthopedic implants and other medical applications [50]. In industry, titanium on a nanoscale is used for its antibacterial activities, such as the long-term storage of wool, which would experience bacterial growth due to the moist environment [51].

Several other metal NPs, such as Ag NPs, Au NPs, Pt NPs, and Cu NPs, and metal oxide NPs, such as MgO and ZnO, exhibit antimicrobial activities [52]. The advantages of TiO_2_ NP employment as an antibacterial agent compared to other types of NPs are its relatively low cost and superior stability of the NPs, while the major disadvantages are the low photocatalytic efficiency of Ti under visible light and the rapid recombination of photogenerated electron-hole pairs, which makes necessary to use metal and non-metal doping, coupling with semiconductors, or modification with graphene oxide in order to enhance the photocatalytic activity [53].

**Figure 3:**
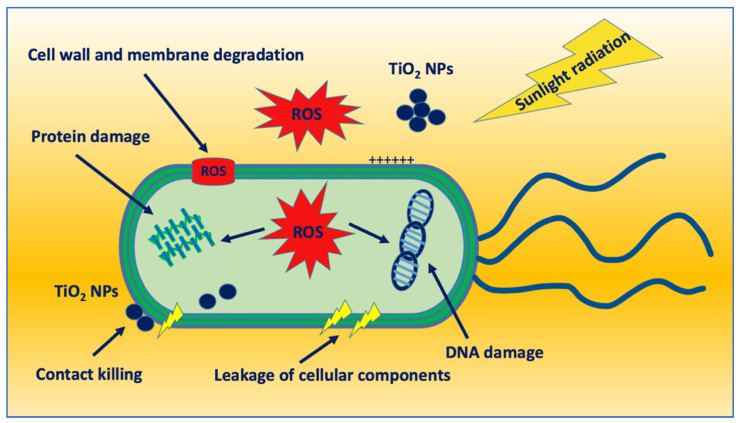
Schematic representation of the antibacterial activity of TiO_2_ NPs.

Since TiO_2_ NPs show a very low dissolution rate, their antibacterial activity is mainly exerted by contact with bacterial surfaces and cell penetration, or ROS production (Figure 3). Studies investigating the membrane interaction of TiO_2_ NPs indicated that the binding of nanoparticles varied with the charge of both the membranes and the nanoparticles. The low charge density of the nanoparticles and/or the lipid bilayers caused fast binding; whereas high negative charge densities of particles and membrane bilayers led to an electrostatic barrier suppressing the binding [54]. The adsorption of nanoparticles onto bacterial surfaces can cause structural destabilization leading to a loss in their integrity and cell death. The effect was slightly different based on the different compositions of cell walls and membranes of Gram-negative and Gram-positive bacteria. The cell wall of Gram-negative bacteria consists of two lipid membranes with an intermediate thin peptidoglycan layer. The outer membrane is rich in negatively-charged lipo-polysaccharides (LPS). The wall of Gram-positive bacteria consists of a single lipid membrane surrounded by a thick peptidoglycan layer and teichoic acids. Khater et al. [55] investigated the effects of TiO_2_ NPs on *Escherichia coli* (Gram-negative) and *Staphylococcus aureus* (Gram-positive) membranes and found that the binding of nanoparticles to the bacterial membranes depolarized the membrane potential of Gram-negative *E. coli* bacteria, but not that of Gram-positive *S. aureus*. The antibacterial effect has been associated with increased membrane permeability and leakage for intracellular proteins, DNA, and ions.

The mode of action of TiO_2_ NPs on Gram-negative bacteria has been shown to be due to osmotic and cell membrane stress caused by the electrostatic binding of TiO_2_ NPs and mechanical membrane disruption [56,57]. A study performed by Leung et al. [58] suggested that the toxicity of TiO_2_ NPs originated from interactions between the nanoparticles and the outer membrane proteins and/or LPS, with mechanical disruption of the cell membrane and possible entry of the nanoparticles into the cell.

The interaction between Gram-positive bacteria and metallic nanoparticles has been highlighted by Jiang et al. [59], who demonstrated that metal oxide NPs, such as Al_2_O_3_, TiO_2_, and ZnO, adsorbed on the structure of cell wall biomolecules, including teichoic acids in vitro, inducing structural changes during contact. Wickman and Rice [60] further concluded that, when lipoteichoic acids simultaneously adhered to peptidoglycan and TiO_2_ NPs, the positively-charged alanine group binds to the surface of negatively charged TiO_2_. Through their interactions, TiO_2_ NPs may cause the alteration of cellular processes as a consequence of structural changes in the cell surface leading to the denaturation of cell wall proteins.

TiO_2_ NPs have been demonstrated to also be able to penetrate bacterial cells as suggested by Leung et al. [58]. Kumar et al. [61] have shown the internalization of TiO_2_ nanoparticles in *Salmonella typhimurium* by both flow cytometry and electron microscopy. Due to its small size and surface charge, nano-titania was able to enter the intracellular compartment of *S.* *Typhimurium*, causing a weak mutagenic response. However, few studies show intracellular penetration of TiO_2_ NPs into bacterial cells; whereas several pieces of evidence indicate an antibacterial effect on surface bacterial structures with subsequent intracellular damage [2,62].

A better antibacterial action of nano-titania has generally attributed to oxidative stress induced by UV light irradiation. The contact of NPs with the bacterial surface leads to the accumulation of free radicals [63] able to induce oxidative stress because of the presence of a wide band gap of 3.2 eV that can trigger the production of high-energy electron-hole pairs when NPs were exposed to UV light with wavelengths of 385 nm or lower [2]. After UV light irradiation, free radicals having high oxidative potential in the presence of oxygen can induce significant effects on the bacterial membranes with a loss in membrane integrity, increase in membrane permeability, marked membrane depolarization, oxidative stress, and membrane lipid peroxidation [2]. In addition, ROS diffusion through damaged bacterial surfaces leads to intracellular stress induction with DNA synthesis alteration, DNA and protein damage, and metabolic enzyme inactivation [62] (Figure 3).

Some studies have demonstrated better antimicrobial performance of TiO_2_ NPs against Gram-positive bacteria [64] compared to Gram-negative bacteria [65]. This seems to be related to the resistance ability of cell wall structures and levels of ROS as well as to the protective function of the outer envelope of Gram-negative bacteria. To provide support against the oxidation produced by ROS, Gram-negative bacteria reinforce the cell membrane by over-expression of genes encoding for enzymes involved in the metabolism of lipids essential for the cell membrane structure [66].

Although bacteria have enzymatic antioxidant defense systems like catalases and superoxide dismutase, able to inhibit lipid peroxidation or neutralize free radicals, when ROS damage exceeds a critical threshold, a self-amplifying process occurs leading to cell death by an irreversible alteration of different essential structures and metabolic pathways [67].

The bactericidal properties of TiO_2_ NPs, or TiO_2_ NP-based nanocomposites, have been extensively investigated (Table 1). Antimicrobial activity has been reported against various bacteria including *E. coli*, *Pseudomonas aeruginosa*, *S. aureus*, *Listeria monocytogenes*, *Salmonella choleraesuis*, and *Vibrio parahaemolyticus* [62,68].

Visible-light illumination is also able to induce photocatalytic activity in TiO_2_ NPs. The bactericidal activity of carbon-containing TiO_2_ nanoparticles under visible-light illumination was evaluated against *Bacillus anthracis.* A significantly-high proportion of bacteria were eliminated following nanoparticle treatment in bacteria-killing experiments. Moreover, macrophage clearance experiments, performed without considerable bacterial killing, showed that photocatalysis of the carbon-TiO_2_ NPs led to a reduction of bacterial resistance against macrophage killing. In addition, 90% of the anthrax lethal toxin content, a major virulence factor of anthrax, was inactivated [79].

The antibacterial effect of TiO_2_ NPs has been extensively investigated in association with other materials in the structured membranes or films and fibers, alone or in combination with antibiotics, mainly for food packaging [80]. TiO_2_ nanoparticle-coated films have been found to have antimicrobial activity at various concentrations under fluorescent and ultraviolet light [81]. A TiO_2_-nanocomposite thin film prepared by an extrusion method followed by UVA light exposure was able to inactivate *Pseudomonas sp*., *Rhodotorula mucilaginosa*, and mesophilic bacteria [82]. TiO_2_ NPs in gelatin-based films also showed excellent antimicrobial activity against *S. aureus* and *E. coli* [83]. According to Gumiero et al. [84], the high-density polyethylene + CaCO_3_ + TiO_2_ composite matrix was able to inhibit lactic acid bacteria and coliforms providing greater retention of the cheese structure. Compared to a TiO_2_ nanoparticle-incorporated film, a sodium alginate film containing functional Au-TiO_2_ nanocomposites improved the antimicrobial activity by 60% and 50% against *S. aureus* and *E. coli*, respectively [85]. TiO_2_-ZnO-MgO mixed oxide nanomaterials are a type of TiO_2_ nano-alloy that has shown good antibacterial properties against *E. coli*, *Salmonella paratyphi*, *S. aureus*, and *L. monocytogenes* [86]. Ansari et al. [70] studied the synthesis and activity of electrospun TiO_2_ nanofibers and demonstrated that they showed more antibacterial effects against gram-negative *P. aeruginosa* cells than gram-positive *S. aureus*. They also demonstrated that TiO_2_ nanofibers inhibited the biofilm formation of methicillin-resistant *S. aureus* (MRSA) and *P. aeruginosa* in a dose-dependent manner. In a recent study, TiO_2_ NPs and red apple pomace, utilized as a potential extraction source to develop a chitosan-based film for packaging, showed a synergistic enhancement in antimicrobial activity as well as antioxidant properties [72].

Salahuddin et al. [87] incorporated TiO_2_ NPs and norfloxacin, an antibiotic to treat bacterial infections, in Polylactic Acid (PLA). Comparison of the antibacterial activity for norfloxacin/PLA and TiO_2_ NPs-norfloxacin/PLA was determined against *S. aureus*, *P. aeruginosa*, *E. coli*, *Salmonella* spp., and *Klebsiella pneumoniae.* Different shapes and concentrations of TiO_2_ NPs were used to assess their effectiveness. Results indicated that the addition of TiO_2_ NPs increased the bacterial inhibition in at least one sample in each bacteria strain. The authors suggested that antibacterial activity can be increased by combining TiO_2_ NPs with an antibiotic, and the release profile of the active ingredients can be adjusted by altering the polymeric material within the system. Gentamicin electrospun with TiO_2_ and poly(ε-caprolactone) to produce a nanofibrous wound dressing showed enhanced inhibition of MRSA *S. aureus* with a synergistic effect between the nanocomposite components [88]. Antibacterial polyurethane/cellulose acetate membranes modified by functionalized TiO_2_ NPs were also evaluated against *E. coli* and MRSA *S. aureus*. Three different NP concentrations were analyzed. After UV irradiation only the higher TiO_2_ concentration (1.5 wt%) exhibited maximum inhibition. The authors concluded that these membranes represent a good material for water purification [89].

Biocompatibility, bioactivity, and wide-ranging antimicrobial activity make nano-titania a potential tool in dental applications. Their ability to provide small pores in bacterial cell walls, leading to broader permeability and cell death, can be used as an antibacterial agent against cariogenic bacteria and biofilms [75]. In addition, the peculiar features of titanium nanoparticles, such as stability, reusability, and photocatalytic activity, are considered useful as a potential alternative in caries prevention. As an example, the incorporation of TiO_2_ NPs into restorative glass ionomer cement significantly improved antibacterial activity together with microhardness and compressive strength, while no decreased adhesion to enamel and dentine was observed [76]. Moreover, experimental adhesives designed to incorporate nitrogen-doped titanium nanoparticles demonstrated superior antibacterial efficacy in dark conditions [77]. Recently, Franzin et al. [90] have evaluated four different formulations containing micro- or nanoparticles of sodium trimetaphosphate as a cement to protect pulp complex. The cement containing sodium trimetaphosphate, ZrO_2_, and TiO_2_ NPs demonstrated the best results in regards to antibacterial activity on *Streptococcus mutans* together with the lowest setting time and high compressive strength. The toxicity against bacteria was attributed to the synergistic and different properties of the cement components, including TiO_2_ NPs producing ROS.

The combination of TiO_2_ NPs with antibiotics has been revealed to be advantageous in improving antibacterial activity. Xu et al. [78] have demonstrated that TiO_2_ NPs at a low level had minimal effects on a wastewater treatment plant, whereas, in combination with tetracycline and erythromycin, the impact of antibiotics was enhanced. This enhanced antibiotic toxicity effect was due to the increasing cell permeability induced by nano-titania. Studies conducted on MRSA *S. aureus* revealed that sub-inhibitory concentrations or different sizes of TiO_2_ NPs were able to increase the antibacterial activities of several antibiotics [73,74]. Moreover, a synergistic effect between TiO_2_ NPs and antibiotics has been demonstrated against multidrug-resistant (MDR) *P. aeruginosa*. It was reported that the presence of antibiotics with nanoparticles increased the concentration of antibiotics at the site of infection and the binding of bacteria to antibiotics [71].

Nano-titania has been used also as a coating for other nanoparticles, such as Ag NPs, or metal or metallic oxide materials, providing an enhancement in antimicrobial activity [69].

## 3. Nanoparticle Bacterial Adaptation

Microbes could have prolonged exposure to nanomaterials because of their deposition in the natural environment and wide use in several fields. This could be responsible for the emergence, prevalence, and spread of microbial adaption/resistance to nanoparticles. The emergence of resistance is due to the selective pressure exerted by nanomaterials together with the peculiar flexibility of the microbial genome. This is particularly relevant for nano-titania because of its wide use and release into the environment [91]. As antibiotic resistance, the resistance to nanomaterials can build up from the abuse of nanomaterials and their ubiquitous occurrence in various environmental matrices. Mechanisms of nanoparticle resistance are different from those of antibiotic resistance because of the peculiar features of nanomaterials. As the antimicrobial activity of nanoparticles depends on their size, shape, and surface properties, the modification of these features by interaction with environmental components influences antibacterial properties, and thereafter the possible development of resistance [92]. Bacterial response to nanomaterials may involve not only full or decreased susceptibility to antibacterial action but also the modulation of virulence factors leading to the development of antibiotic resistance or increased virulence [3].

**Figure 4:**
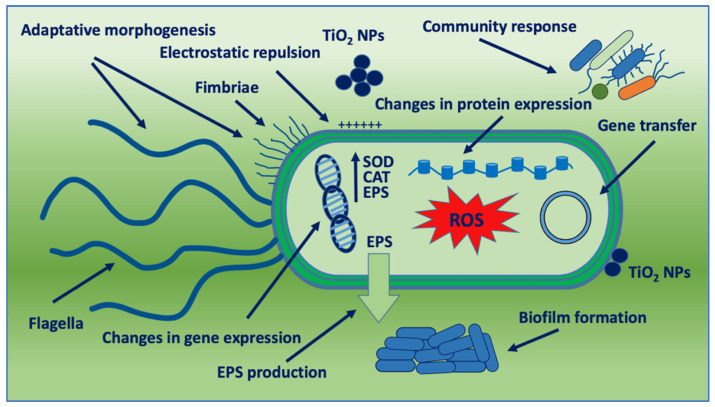
Schematic representation of bacterial adaptation mechanisms against TiO_2_ NPs. SOD: SuperOxide Dismutase; CAT: Catalase; and EPS: Extracellular Polymeric Substance.

Reported microbial resistance mechanisms to nanoparticles include efflux pumps, electrostatic repulsion, alteration of morphology, biofilm formation, extracellular matrices, gene transfer, metabolic responses, and mutations [93] (Figure 4). These mechanisms are generated by the bacterial response to nanoparticle-induced stress and can be observed singly or collectively, and in dependence of single cells or bacterial communities and environmental conditions. In addition, acute and chronic cell or tissue exposure to nanoparticles can induce changes that influence bacterial opportunistic mechanisms favoring microbial virulence.

Studies on TiO_2_ NP adaptation are displayed in Table 2. Apart from efflux systems, which do not seem to be involved in bacterial adaptation, the adaptation responses to the TiO_2_ NP challenge will be illustrated below as nanoparticle impact on bacterial fitness. Efflux pumps participate in the development of resistance to ion release from metal nanoparticles under non-bactericidal concentrations, but TiO_2_ NPs do not seem to release ions because of their very low dissolution rate and high stability [83].

### 3.1. Electrostatic Repulsion and Charge Modification

Some resistance mechanisms to nanoparticles involve electrostatic repulsion. Bacteria are able to regulate the electrical charge of their surface, which allows them to repel nanoparticles with different types of charge on their surface. The antimicrobial activity of the nanoparticles in some cases can be due to the interaction of nanoparticle charge with the electric charge present on the surfaces of the bacteria [119], but some bacteria acquire mechanisms allowing resistance to the charge of nanoparticles [120].

Planchon et al. [94] studied the interaction between *E. coli* and TiO_2_ NPs in natural and artificial waters and found that pH and dispersion status influenced the contact between the NPs and *E. coli*. TiO_2_ NP toxicity seemed to be due to the aggregation status of the nanoparticles and the physiological state of *E. coli* at different pH. Compared to at pH 8, a better bacterial physiological state with better resistance to nanoparticle toxic effect was observed at pH 5, despite a stronger interaction between the cells and nanoparticles. At the same time, a bacterial subpopulation, apparently non-interacting with the nanoparticles, was found. Such heterogeneities in cell populations could be related to a strategy of the colony where some bacteria try to adsorb all the contaminants in the solution, including TiO_2_ NPs, favoring bacterial resistance. Similar results were obtained from our study on the interaction between *L. monocytogenes* and non-UV irradiated TiO_2_ NPs. The influence of nanoparticles depended on nanoparticle concentrations and the biofilm-forming capability of the bacterial strains. Close contact of bacteria with TiO_2_ NPs was extensively observed without decreased listeria vitality. A pH of 5.5 seemed to favor the close adhesion of nano-titania to bacterial cell surfaces, probably because of increased bacterial hydrophobicity. Bacterial-TiO_2_ NP interaction led to an increased biofilm formation. It appeared that *L. monocytogenes* exploited the large agglomerates of TiO_2_ particles to massively adhere and promote bacterial aggregation and biofilm production, in order to prolong their survival and dissemination [95].

It is known that the hydrophobicity of both bacteria and surfaces could play a role in their initial interaction with each other depending on several factors such as temperature, nutritional content, or bacterial features [121]. A higher interaction between nanoparticles and bacteria is expected to enhance the toxic effects of the nanoparticles. The absence of UV light irradiation during the interaction at acidic pH can explain the low or no significant toxicity of TiO_2_ NPs. On the contrary, nanoparticle adsorption on bacterial surfaces seems to promote bacterial survival strategies.

### 3.2. Adaptative Morphogenesis

It has been reported that nano-resistance begins with changes in the shape of bacteria and modulation of membrane protein expression [122]. Generally, adaptative morphogenesis was observed in chronic exposure to nanoparticles. Repeated exposure of a commensal *E. coli* strain to a low dose of nano-titania for 400 days in the dark led to filamentation, thickening of the cell wall, and biofilm formation, accompanied by decreased sensitivity to oxidative stress and multiple antibiotics. Enhanced bacterial motility was observed with flagellar assembly, and fimbria biosynthesis increased. These adaptive traits were associated with increased pathogenicity, as confirmed by a higher death rate of the macrophages in vitro and more severe bacterial infection in mice *in vivo*. The adaptive evolution was attributed to free-radical production by nano-titania in the dark. This study is one of only a few evaluating free radical production in the dark. TiO_2_ NPs were able to generate low levels of ROS, specifically hydroxyl radicals, in dark conditions such as in the gut, probably due to nanoparticle surface defects, such as oxygen vacancies. This oxidative stress induced a commensal-to-pathogen transition of *E. coli*, raising concern mainly for intestinal microbiota [96].

*E. coli* showed also an adaptative response to palladium oxide-modified nitrogen-doped TiO_2_ (TiON/PdO) under UV light irradiation [97]. After repeated exposure to photo-disinfection, *E. coli* adapted its response by regulation of chemotaxis and flagellar assembly followed by increased superoxide radical degradation. After photocatalysis of TiON/PdO nanoparticles, a mutant strain was obtained showing a small colony size and irregular margin morphology. Metabolic processes of the mutant, such as oxidative phosphorylation, TCA cycle, glycolysis, pyruvate, fatty acid, and glutathione, were decreased. Motility was enhanced through the up-regulation of genes in the flagellar assembly pathway during the stress response. *E. coli* response to photocatalysis was adapted through an enhanced ability for superoxide radical degradation.

Following the discovery that biofilms formed from activated sludge exposed to 5 and 50 mg/L nano-titania in the dark had increased biomass and selectively enriched pathogens, Zhu et al. [98] examined the protein response and protein phosphorylation modification of *E. coli* K12 exposed to nano-titania. Using the integrative systems biology analyses of proteomics and phosphoproteomics, they demonstrated that *E. coli* cultivated with TiO_2_ NPs up-regulated iron acquisition and regulated protein phosphorylation states associated with transcription, translation, and biofilm formation. Bacteria showed increased siderophores and exopolysaccharide content together with enhanced resistance to transcriptional inhibitory antibiotics. Some up-regulated proteins were associated with increased curli production and cellulose biosynthesis, which are important components of the extracellular matrix of *E. coli* supporting biofilm formation. *E. coli* was therefore shown to adapt to sublethal TiO_2_ NP concentrations by adaptative morphogenesis leading to bacterial survival by promoting biofilm formation.

### 3.3. Community Response

The bactericidal activity of TiO_2_ NPs is mainly due to ROS generation during the interaction with bacteria that develop different strategies to counteract this challenge [123]. Bacterial adaptation to TiO_2_ NPs producing ROS was observed mainly in multispecies microbial aggregates under chronic nanoparticle exposure.

Engineered TiO_2_ NPs are released into biological wastewater treatment plants and are recognized as environmental stressors. Mathur et al. [99] have demonstrated that a consortium of different bacteria in wastewater is able to reduce damage from oxidative stress by TiO_2_ NPs. The viability of the consortium is higher than that of single isolates. In particular, some bacteria of the consortium, such as *Exiguobacterium acetylicum* and *Pseudomonas nitroreducens*, having a higher capacity to produce SOD enzyme, contributing to the survival of other bacteria. Extracellular polymeric substance (EPS) production was also most expressed in the bacterial consortium compared to single bacteria. Capsular EPS provides a defense against the attachment of TiO_2_ NPs and ROS diffusion to the bacterial cells preventing membrane integrity loss. In addition, enhanced release of EPS corresponded to increased biofilm production, with most of the nanoparticles and ROS able to access bacterial cell surfaces. Thus, the consortium of cells was shown to have better abilities to counteract the toxic effects of TiO_2_ NPs, whilst also maintaining the ability to reduce organics in sewage.

Periphytic biofilm, a typical autotropic multispecies microbial aggregate, also showed adaptation to TiO_2_ NP impact. While there is no evident toxic effect of nanoparticle exposure on periphytic biofilm in terms of biomass, chlorophyll content, and ATPase activity, the microbial communities were protected from ROS production and accumulation. Moreover, periphytic biofilms changed their community composition in the presence of TiO_2_ NPs by increasing the relative abundance of phototrophic and high-nutrient metabolic microorganisms [100].

Changes in the bacterial abundance in multicellular bacterial communities were found also in studies on TiO_2_ impact on the growth and activity of bacterial communities of three Swedish lakes. Exposure to different concentrations of TiO_2_ NPs, and in the presence of particular environmental conditions that make nanoparticles stable, significantly reduced bacterial abundance. Despite the reduction of bacterial abundance following nanoparticle exposure, the overall bacterial activity did not, in most cases, change significantly, which was due to a strongly enhanced activity per cell in the higher TiO_2_ NP concentration exposure group. This indicated the presence of bacterial groups that are more resistant to TiO_2_ NP toxicity or are even stimulated in the presence of TiO_2_ NPs [101].

Perturbation of microbial communities was reported also in soil bacteria exposed to TiO_2_ NPs over time. Short-term TiO_2_ NP exposure revealed significant effects on enzyme activity and bacterial community structure and composition in clay soil with high organic matter. Response alterations were observed in the taxa belonging to Acidobacteria and Verrucomicrobia, and functional pathways related to carbohydrates degradation. As exposure time increased, the bacterial community recovered after long-term exposure of 60 days, suggesting that the bacterial evolution and adaptation could overcome the TiO_2_ NP selection after long-term exposure [102].

### 3.4. Metabolic Response

The presence of environmental stressors, such as nanoparticles, can influence bacterial gene expression and facilitate resistance mechanisms. The release of TiO_2_ NPs into biological wastewater treatment plants has drawn significant attention because microorganisms used for pollutant removal are potentially threatened by the TiO_2_ NPs due to their biotoxicity. A study conducted in a chemostat reactor exploring the behaviour of ammonia oxidizer bacteria under chronic TiO_2_ NP exposure indicated that *Nitrosomonas europea* was able to adapt to the TiO_2_ stressor. After 40 days of incubation, *N. europea* cultures appeared to recover cell growth inhibition, membrane integrity, nitrification rate, and ammonia monooxygenase activity. The recovery capacities of the bacteria were associated with the activation of several metabolic activities, such as processes involved in membrane repair and metabolic and stress-defense pathways. Changes in these metabolic processes induced cellular adaptation and recovery, providing the selection of resistant bacterial cells [103].

The impact of nano-titania on the physiological function of *Shewanella oneidensis*, a metal reducer bacterium, has been evaluated. *S. oneidensis* secretes flavin mononucleotide that was rapidly converted into riboflavin that transforms metals and serves as a method of respiration for *S. oneidensis* in limited oxygen content. After exposure to varying concentrations and types of TiO_2_ NPs, minimal changes in vitality were observed, whereas significant changes in bacterial growth, biofilm formation, and riboflavin secretion of *S. oneidensis* occurred. These changes were the result of the proximity of the nanoparticles causing altered gene expression, which influenced bacterial activities such as biofilm growth and riboflavin secretion. Bacterial growth showed a dose-dependent increase whereas biofilm production revealed a slower biofilm growth. In addition, extracellular riboflavin increased as a function of nanoparticle concentration. These metabolic changes were due to the modification of gene expression induced by the TiO_2_ NPs. In particular, increased expression of riboflavin correlated with omcA expression, which encodes for an outer membrane c-type cytochrome that plays a small role as a terminal reductase for metals. This alteration indicated that *S. oneidensis* flavin secretion is activated as a response to a system stressor [104].

An unexpected TiO_2_ NP resistance phenotype was found in a study evaluating the interaction of nanoparticles with an LPS-truncated *E. coli* K12 mutant. The exposure of bacteria carrying this core-free LPS to nanoparticles in the dark increased the action of nanoparticles, with the stripping of outer membranes, increased osmotic stress, and efficient vesicle-facilitated release of damaged membrane components. In addition, vesicles were observed acting as electrostatic baits for TiO_2_ NPs, mitigating TiO_2_ NP toxicity. Surprisingly, the TiO_2_ NP activity on the altered LPS structure favored a further membrane destabilization that seemed to be able to generate an antagonistic response to nanoparticles [106].

In some cases, a stress response to nanoparticles could favor antibiotic activity, as found during the evaluation of the impact of TiO_2_ NPs on the activity of antimicrobials, quorum sensing (QS), and efflux pump genes expression in MDR *P.aeruginosa* isolates. Nano-titania not only exerted antibacterial activity against *P. aeruginosa* and high reduction of biofilm formation but, when used alone or in combination with antibiotics, provided a significant down-regulation of the efflux pump genes (MexY, MexB, and MexA) and QS-regulated genes (lasR, lasI, rhll, rhlR, pqsA, and pqsR). This effect allowed for a better response to antibiotics against MDR *P. aeruginosa* [105]. Nevertheless, it cannot be ruled out that photoactivated TiO_2_ NPs could induce bacterial antibiotic tolerance that could evolve into resistance. It was observed that the *E. coli* DH5α strain treated with lethal photo-activation showed bacterial stress responses that improve antibiotic tolerance by several mechanisms, such as efflux pumps, biofilm formation, and increased mutation rates. The bacteria with higher antibiotic tolerance could evolve into antibiotic resistance faster with subsequent antibiotic selection [124].

### 3.5. Gene Transfer

Nanoparticles, acting as environmental stressors, can cause a spontaneous rise in mutation and trigger genome plasticity, which can greatly facilitate resistance to antimicrobial agents and the evolution of strains with increased fitness. By altering bacterial physiology, and especially competence, NPs may influence the dissemination of antibiotic resistance in bacteria. Nano-titania is able to significantly modify the transformation efficiency of *Bacillus subtilis* in biofilm growth conditions [107]. Transformation is defined as the uptake of foreign DNA and its subsequent integration into the bacterial chromosome or replication as an independent plasmid. The first step in the transformation is the “competence”, which involves the take up of DNA through the bacterial surface and then the complete entry of DNA in the cell. After *B. subtilis* exposure to nano-titania, the competence appeared to be significantly decreased. Two oligopeptide ABC transporters, OppABCDF and AppDFABC, are differentially expressed in response to nanoparticles. The Opp and App transporters are responsible for the import of the extracellular peptide factors, which initiate the competence process. These processes involved in the induction of competence were affected as a consequence of a physiological adaptation.

Regarding the spread of antibiotic resistance genes mediated by nanoparticles, it was observed that at various nanomaterial concentrations, bacterial density, matting time, and matting temperature, nano-titania can significantly promote the conjugation of the RP4 plasmid in *E. coli*. A mathematical model to quantitatively describe the conjugation process and evaluate the effects of TiO_2_ NPs on the spread of antibiotic resistance genes revealed that the nanoparticles inhibited bacterial growth and promoted conjugation simultaneously with a potential environmental risk [108]. The spread of antibiotic resistance genes has been demonstrated also by phage infection mediated by TiO_2_ NPs, which exhibited the ability to promote bacteriophage attachment on cell surfaces as the constructed phage gM13 that infects *E. coli* TG1 strain. Increased membrane permeability induced by nano-titania appeared to facilitate the infectious entry of phage gM13 into periplasmic space. Following TiO_2_ NP photoexcitation, extracellular ROS production was shown to facilitate phage entry by increasing the peroxidation of phospholipids and damaging the integrity of the bacterial membrane. Moreover, the expression of pilus-related genes was improved when *E. coli* TG1 was exposed to TiO_2_ NPs and photoexcitation. This enhanced pili-related gene expression promoted the synthesis of bacterial pili, thereby increasing the phage invasion sites and improving the transduction efficiency [109].

### 3.6. Impact on Intestinal Microbiota

TiO_2_ NPs introduced while consuming food can exert an influence on the human microbiome. During their passage through the small intestine, they come in contact with proteins and peptides that can interact with the NPs forming agglomerates, as well as changing their charge [125]. Moreover, the contact of TiO_2_ NPs with commensal bacteria can influence the resident microbiota by inhibiting the growth and activity of gastrointestinal bacteria, mainly in bacteria of the probiotic type. The bacterial communities composing the intestinal microbiota can develop adaptative strategies to survive, such as biofilm formation or metabolic regulation [110]. Taylor et al. [111], by using three different nanoparticles including nano-titania in a colon model, demonstrated changes in multiple characteristics of bacteria phenotypes, such as hydrophobicity, the sugar content of EPS, electrophoretic mobility, and the production of short-chain fatty acids. The most relevant phenotypic transformation induced by TiO_2_ NP exposure was the hydrophobicity leading to an increased trend in biofilm formation. Pinget et al. [112], after oral administration of TiO_2_ NPs, reported changes in the release of bacterial metabolites of commensal bacteria *in vivo*, and biofilm promotion in vitro–although minimum NP impact on the composition of gastrointestinal microbiota in mice was found.

Metabolomic and proteomic responses of *E. coli* to nano-titania stress observed by Planchon et al. [115] demonstrated differences between bacteria fully covered with TiO_2_ NPs and the population that remained free from nanoparticles. Several proteins appeared down-regulated whereas the proteins associated with energy metabolism were up-regulated. The proteins most affected by the exposure to nanoparticles are those associated with the integrity of the membrane, together with proteins involved in the stress response, DNA protection during starvation, or those which promote protein folding. The up-regulated proteins were involved in energy metabolism, especially glycolysis and the TCA cycle. Moreover, the synthesis of amino acids or proteins involved in biosynthetic processes was modified. The authors concluded that the exposure of *E. coli* cells to nano-titania led to a heterogeneous response with part of the bacterial population able to adapt to TiO_2_ NP stress and survive, while the remainder died because they were unable to adapt to this stress.

Waller et al. [113] demonstrated that bacterial exposure to TiO_2_ NPs in an in vitro human colon reactor model caused changes to the composition of the microorganisms, as well as lowering the colonic pH. The addition of industrial-grade and food-grade TiO_2_ NPs to the colon model resulted in a bacterial response linked to the microbial composition and phenotypic and biochemical changes with a reduced transition of the microbial community from Proteobacteria abundance to Firmicutes. Moreover, a reduced system pH and conductivity, with probable disruption of the anaerobic digestive process, was observed. These alterations representing bacterial community adaptation to TiO_2_ NPs can lead to the potential onset of deleterious conditions over continuous, long-term exposure.

At the same time, literature data are available that reveal a limited influence or no significant direct impact of TiO_2_ NPs on human gastrointestinal microbiota. Low TiO_2_ NP concentrations, equivalent to those found in chewing gums and candies, showed low effects on the intestinal bacterial community known as microbial ecosystem therapeutic-1 (MET-1), and no changes in gas production and fatty acid methyl ester profiles, suggesting no significant influence on bacterial metabolism and microbiota composition [116]. Similar results were obtained by Agans et al. [114], using an in vitro Human Gut Simulator (HGS). Cultures exposed to TiO_2_ NPs displayed only a modest reduction in microbial community density with no impact on community diversity and evenness, even though the NPs were found to loosely interact with microbial cells.

### 3.7. Impact on Bacteria-Cell Interaction

TiO_2_ NP impact on bacterial virulence also plays a role in bacteria-cell interactions. Cells or tissues interacting with TiO_2_ NPs can undergo changes that can make them more susceptible to bacterial adhesion or invasion. It was reported that TiO_2_ particles, that exhibit no sign of toxicity, were able to perturb the cholesterol gradient on HeLa cell membranes [117]. In particular, cholesterol in the inner plasma membrane leaflet was reduced, while there was an increased amount of cholesterol in the outer plasma membrane leaflet. The enhanced asymmetry in the cholesterol distribution caused an increase in *S. aureus* infection on HeLa cells, as *S. aureus* requires cholesterol for proper membrane attachment and virulence. Cell exposure to low TiO_2_ NP concentrations led to the up-regulation of the cholesterol transporter proteins that facilitate the transport of cholesterol across membranes. Thus, nano-titania, rather than preventing bacterial cell infection, promoted bacterial infectivity by regulation of cellular genes that modify the structural features of membranes.

*L. monocytogenes* also showed increased invasiveness in intestinal cells pretreated with nano-titania at low doses. In a study performed by the authors of this review, the in vitro exposure of human intestinal cells to nonactivated TiO_2_ NPs before *L. monocytogenes* infection significantly increased the efficiency of bacterial invasion and survival. Pretreatment of HT-29 cells with 1 μg/cm^2^ of TiO_2_ NPs, comparable to the real amount of TiO_2_ ingestion through food, induced higher invasiveness compared to untreated cells. Cytoskeletal changes, probably induced by TiO_2_ NP treatment, enhanced bacterial internalization. In addition, increased bacterial entry led to higher intracellular bacterial survival [118].

In these cases, TiO_2_ NPs are not intrinsically anti-bacterial and, when ingested by cells, they do not exert cellular toxicity by the generation of free radicals or chemical damage to cell structures. Nanoparticles at low concentrations and non-UV irradiated act in a subtle manner inducing cell membrane or intracellular changes leading to an increased bacterial invasion.

## 4. Conclusions

Due to the large use of nano-titania and its application as an antibacterial agent, bacterial adaptation to this nanomaterial is expected to increase in the future under nano-titania environmental pressure. Although TiO_2_ NPs have been considered for a long time as a safe material, and recently as a promising tool to counteract antibiotic resistance, today we know that they can induce several alterations in biological systems. The negative impact of TiO_2_ NPs on bacterial cells is increasingly recognized and is leading to concerns regarding their biosafety and biosecurity. Combating microbial resistance by using nano-titania–especially in combination with antibiotics–could lead to transiently antibiotic tolerance improvement with the selection of tolerant bacteria that could rapidly evolve antibiotic resistance under exposure to sublethal doses of antibiotics.

Bacterial adaptation to nano-titania is more pronounced in multicellular bacterial communities that constantly adapt their population fitness to unfavorable environments causing stress. Multispecies microbial aggregates can protect cells because of intra- and inter-specific networks that are beneficial to ROS detoxification. However, chronic exposure to TiO_2_ NPs seems to lead to the selection of bacteria populations in microbial communities with a different pattern of tolerance to the oxidative stress induced by the nanoparticles. These populations contribute to the formation of persister cells that emerge following stress response by activating stress signaling pathways. Once persisters are selected from the original population, antibiotic treatment failure occurs, because of their high tolerance to antibiotics and other stress factors. Bacterial adaptation to nano-titania in some cases could result in an advantage for human activities, such as during bacterial recovery by the cooperation of microorganisms belonging to consortium systems used to reduce organics in sewage. However, it cannot be ruled out that chronic exposure to nano-titania could induce different and detrimental responses over time. Particular attention must be paid when photobiocatalysis is used to improve contaminant removal efficiency. The impact of TiO_2_ NPs on bacteria residing in the human gut must also be considered. Microbiota showed bacterial responses leading to changes in the composition of the microorganism population and environmental conditions indicating bacterial adaptation to the nanoparticles. The possible bacterial transition from commensal to pathogen must also be carefully investigated. Finally, TiO_2_ NP modifications of cell and/or tissue pathways representing the target of bacterial virulence can induce an indirect effect on bacterial adaptation with an improvement in bacterial infectivity.

Considering the high doses of TiO_2_ NPs to which people are exposed daily, it is necessary to systematically assess their pathophysiological effect on bacteria. To minimize detrimental effects on TiO_2_ NP applications, it would be desirable that the relationship between TiO_2_ NP properties and microorganism adaptation is extensively investigated, particularly from the perspective of long-term exposure.

## Figures and Tables

**Table 1:** TiO_2_ NP antibacterial activity.

Material	Bacteria	Reference
TiO_2_ NPs	*Escherichia coli*	[62,68,69]
	*Pseudomonas aeruginosa*	[62,68,69,70,71]
	*Staphylococcus aureus*	[62,68,69,70,72,73,74]
	*Listeria monocytogenes*	[62,68]
	*Salmonella choleraesuis*	[62,68]
	*Vibrio parahaemolyticus*	[62,68]
	*Streptococcus mutans*	[75,76,77]
	Bacterial community of nitrification system	[78]
Carbon-containing TiO_2_ NPs	*Bacillus anthracis* *Bacillus subtilis* *Bacillus cereus* *Bacillus thuringiensis*	[79]
Low-density polyethylene/AgNPs/TiO_2_ NPs	*Escherichia coli* *Staphylococcus aureus*	[80]
Chitosan-TiO_2_ NPs/Red apple pomace extract	*Escherichia coli* *Staphylococcus aureus*	[72]
Low-density polyethylene/AgNPs + CuNPs/TiO_2_ NPs	*Escherichia coli* *Listeria monocytogenes*	[80]
Low-density polyethylene/TiO_2_ NPs	*Escherichia coli*	[81]
	*Pseudomonas* spp. *Rhodotorula mucilaginosa*	[82]
Bovine gelatin films/TiO_2_ NPs	*Escherichia coli* *S* *taphylococcus aureus*	[83]
High density polyethylene/CaCO_3_/TiO_2_ NPs	Lactic acid bacteria Coliforms	[84]
Sodium alginate/Au-TiO_2_ NPs	*Staphylococcus aureus* *Escherichia coli*	[85]
TiO_2_-ZnO-MgO NPs	*Escherichia coli* *Salmonella paratyphi* *Staphylococcus aureus* *Listeria monocytogenes*	[86]
Poly(lactic acid)/TiO_2_ NPs	*Staphylococcus aureus* *Pseudomonas aeruginosa* *Escherichia coli* *Salmonella* spp. *Klebsiella pneumoniae*	[87]
Poly(ε-caprolactone)/TiO_2_ NPs	*Staphylococcus aureus*	[88]
Polyurethane/cellulose acetate/TiO_2_ NPs	*Escherichia coli* *Staphylococcus aureus*	[89]
Nitrogen-doped TiO_2_ NPs	*Streptococcus mutans*	[75]
Sodium trimetaphosphate/TiO_2_ NPs	*Streptococcus mutans* *Lactobacillus casei* *Actinomyces israelii* *Enterococcus faecalis*	[90]

**Table 2:** TiO_2_ NP bacteria adaptation.

Adaptation Mechanism	Bacteria	Reference
Electrostatic repulsion and charge modification	*Escherichia coli*	[94]
	*Listeria monocytogenes*	[95]
Adaptative morphogenesis	*Escherichia coli*	[96,97]
	*Escherichia coli K12*	[98]
Community response	*Exiguobacterium acetylicum*	[99]
	*Pseudomonas nitroreducens*	
	Periphytic biofilm	[100]
	Freshwater bacteria	[101]
	Proteobacteria Acidobacteria Actinobacteria Verrucomicrobia	[102]
Metabolic response	*Nitrosomonas europaea*	[103]
	*Shewanella oneidensis*	[104]
	*Pseudomonas aeruginosa*	[105]
	Lipopolysaccharide-truncated *Escherichia coli* K12 mutant	[106]
Gene transfer	*Bacillus subtilis*	[107]
	*Escherichia coli*	[108]
	*Escherichia coli* TG1	[109]
Microbiota modification	Human Microbiota	[110]
	Human Microbiota	[111]
	Gut Microbiota	[112,113,114]
	*Escherichia coli*	[115]
	Human gut bacterial community MET-1	[116]
Increased bacteria-cell interactions	*Staphylococcus aureus*	[117]
	*Listeria monocytogenes*	[118]

## Data Availability

Not applicable.

## References

[1] Baranowska-Wójcik E., Szwajgier D., Oleszczuk P., Winiarska-Mieczan A. (2020). Effects of Titanium Dioxide nanoparticles exposure on human health—A review. Biol. Trace Elem. Res..

[2] De Dicastillo C.L., Correa M.G., Martínez F.B., Streitt C., Galotto M.J. (2020). Antimicrobial Effect of Titanium Dioxide Nanoparticles. Antimicrobial Resistance—A One Health Perspective.

[3] Zhang C., Sun R., Xia T. (2020). Adaption/resistance to antimicrobial nanoparticles: Will it be a problem?. Nano Today.

[4] Li J.G., Ishigaki T., Sun X. (2007). Anatase, brookite, and rutile nanocrystals via redox reactions under mild hydrothermal conditions:  phase-selective synthesis and physicochemical properties. J. Phys. Chem. C.

[5] Warheit D.B., Brown S.C. (2019). What is the impact of surface modifications and particle size on commercial titanium dioxide particle samples? A review of in vivo pulmonary and oral toxicity studies. Toxicol. Lett..

[6] Di Paola A., Bellardita M., Palmisano L. (2013). Brookite, the least known TiO_2_ photocatalyst. Catalysts.

[7] Bolis V., Busco C., Ciarletta M., Distasi C., Erriquez J., Fenoglio I., Livraghi S., Morel S. (2012). Hydrophilic/hydrophobic features of TiO_2_ nanoparticles as a function of crystal phase, surface area and coating, in relation to their potential toxicity in peripheral nervous system. J. Colloid Interface Sci..

[8] Gobi R., Ravichandiran P., Babu R.S., Yoo D.J. (2021). Biopolymer and synthetic polymer-based nanocomposites in wound dressing applications: A review. Polymers.

[9] Moaveni P., Farahani H.A., Maroufi K. (2011). Effect of TiO_2_ nanoparticles spraying on barley (*Hordeum vulgare* L.) under field condition. Adv. Environ. Biol..

[10] Irshad M.A., ur Rehman M.Z., Anwar-ul-Haq M., Rizwan M., Nawaz R., Shakoor M.B., Wijaya L., Alyemeni M.N., Ahmad P., Ali S. (2021). Effect of green and chemically synthesized titanium dioxide nanoparticles on cadmium accumulation in wheat grains and potential dietary health risk: A field investigation. J. Hazard. Mater..

[11] Khattak A., Ullah F., Shinwari Z.K., Mehmood S. (2021). The effect of titanium dioxide nanoparticles and salicylic acid on growth and biodiesel production potential of sunflower (*Helianthus annuus* L.) under water stress. Pak. J. Bot..

[12] Korösi L., Bognár B., Czégény G., Lauciello S. (2022). Phase-selective synthesis of anatase and rutile TiO_2_ nanocrystals and their impacts on grapevine leaves: Accumulation of mineral nutrients and triggering the plant defense. Nanomaterials.

[13] Amalraj A., Pius A. (2015). Photocatalytic degradation of monocrotophos and chlorpyrifos inaqueous solution using TiO_2_ under UV radiation. J. Water Process Eng..

[14] Dréno B., Alexis A., Chuberre B., Marinovich M. (2019). Safety of titanium dioxide nanoparticles in cosmetics. J. Eur. Acad. Dermatol. Venereol..

[15] Tyner K.M., Wokovich A.M., Godar D.E., Doub W.H., Sadrieh N. (2011). The state of nano-sized titanium dioxide (TiO_2_) may affect sunscreen performance. Int. J. Cosmet. Sci..

[16] Center For Food Safety “Nanotechnology in Food”. https://www.centerforfoodsafety.org/nanotechnology-in-food.

[17] Weir P., Westerhoff L., Fabricius K., von Goetz H.N. (2012). Titanium dioxide nanoparticles in food and personal care products. Environ. Sci. Technol..

[18] Chen X.X., Cheng B., Yang Y.X., Cao A., Liu J.H., Du L.J., Liu Y., Zhao Y., Wang H. (2013). Characterization and preliminary toxicity assay of nano-titanium dioxide additive in sugar-coated chewing gum. Small.

[19] Peters R.J.B., van Bemmel G., Herrera-Rivera Z., Helsper H.P.F.G., Marvin H.J.P., Weigel S., Tromp P.C., Oomen A.G., Rietveld A.G., Bouwmeester H. (2014). Characterization of titanium dioxide nanoparticles in food products: Analytical methods to define nanoparticles. J. Agric. Food Chem..

[20] Verleysen E., Waegeneers N., Brassinne F., De Vos S., Jimenez I.O., Mathioudaki S., Mast J. (2020). Physicochemical characterization of the pristine E171 food additive by standardized and validated methods. Nanomaterials.

[21] E.C (2011). Commission recommendation of 18 October 2011 on the definition of nanomaterial. O. J. Eur. Union.

[22] Dudefoi W., Terrisse H., Popa A.F., Gautron E., Humbert B., Ropers M.H. (2018). Evaluation of the content of TiO_2_ nanoparticles in the coatings of chewing gums. Food Addit. Contam. Part A.

[23] EFSA (2004). Opinion of the Scientific Panel on food additives, flavourings, processing aids and materials in contact with food (AFC) on Titanium dioxide. EFSA J..

[24] Setyawati M.I., Zhao Z., Ng K.W. (2020). Transformation of nanomaterials and its implications in gut nanotoxicology. Small.

[25] EFSA FAF Panel (EFSA Panel on Food Additives and Flavourings) (2021). Scientific Opinion on the safety assessment of titanium dioxide (E171) as a food additive. EFSA J..

[26] Falahati M., Attar F., Sharifi M., Haertl´e T., Berret J.F., Khan R.H., Saboury A.A. (2019). A health concern regarding the protein corona, aggregation and disaggregation. Biochim. Biophys. Acta Gen. Subj..

[27] Moradi M., Razavi R., Omer A.K., Farhangfar A., McClements D.J. (2022). Interactions between nanoparticle-based food additives and other food ingredients: A review of current knowledge. Trends Food Sci. Technol..

[28] Bing J., Xiao X., McClements D.J., Biao Y., Chongjiang C. (2021). Protein corona formation around inorganic nanoparticles: Food plant proteins-TiO_2_ nanoparticle interactions. Food Hydrocoll..

[29] Cao X., Han Y., Li F., Li Z., McClements D.J., He L., Decker E.A., Xing B., Xiao H. (2019). Impact of protein-nanoparticle interactions on gastrointestinal fate of ingested nanoparticles: Not just simple protein corona effects. NanoImpact.

[30] Wang Y., Li M., Xu X., Tang W., Xiong L., Sun Q. (2019). Formation of protein corona on nanoparticles with digestive enzymes in simulated gastrointestinal fluids. J. Agric. Food Chem..

[31] Coreas R., Cao X., DeLoid G.M., Demokritou P., Zhong W. (2020). Lipid and protein corona of food-grade TiO_2_ nanoparticles in simulated gastrointestinal digestion. NanoImpact.

[32] McClements D.J., Xiao H., Demokritou P. (2017). Physicochemical and colloidal aspects of food matrix effects on gastrointestinal fate of ingested inorganic nanoparticles. Adv. Colloid Interface Sci..

[33] Han H.Y., Yang M.J., Yoon C., Lee G.H., Kim D.W., Kim T.W., Kwak M., Heo M.B., Lee T.G., Kim S. (2021). Toxicity of orally administered food-grade titanium dioxide nanoparticles. J. Appl. Toxicol..

[34] Bettini S., Boutet-Robinet E., Cartier C., Coméra C., Gaultier E., Dupuy J., Naud N., Taché S., Grysan P., Reguer S. (2017). Food-grade TiO_2_ impairs intestinal and systemic immune homeostasis, initiates preneoplastic lesions and promotes aberrant crypt development in the rat colon. Sci. Rep..

[35] Talamini L., Gimondi S., Violatto M.B., Fiordaliso F., Pedica F., Lan Tran N., Sitia G., Aureli F., Raggi A., Nelissen I. (2019). Repeated administration of the food additive E171 to mice results in accumulation in intestine and liver and promotes an inflammatory status. Nanotoxicology.

[36] Cui Y., Liu H., Ze Y., Zengli Z., Hu Y., Cheng Z., Cheng J., Hu R., Gao G., Wang L. (2012). Gene expression in liver injury caused by long-term exposure to titanium dioxide nanoparticles in mice. Toxicol. Sci..

[37] Blevins L.K., Crawford R.B., Bach A., Rizzo M.D., Zhou J., Henriquez J.E., Khan D.M.I.O., Sermet S., Arnold L.L., Pennington K.L. (2019). Evaluation of immunologic and intestinal effects in rats administered an E 171-containing diet, a food grade titanium dioxide (TiO_2_). Food Chem. Toxicol..

[38] Kaegi R., Ulrich A., Sinnet B., Vonbank R., Wichser A., Zuleeg S., Simmler H., Brunner S., Vonmont H., Burkhardt M. (2008). Synthetic TiO_2_ nanoparticle emission from exterior facades into the aquatic environment. Environ. Pollut..

[39] Windler L., Lorenz C., von Goetz N., Hungerbühler K., Amberg M., Heuberger M., Nowack B. (2012). Release of titanium dioxide from textiles during washing. Environ. Sci. Technol..

[40] Vidmar J., Zuliani T., Milačič R., Ščančar J. (2022). Following the occurrence and origin of titanium dioxide nanoparticles in the Sava river by single particle ICP-MS. Water.

[41] Yuan S., Huang J., Jiang X., Huang Y., Zhu X., Cai Z. (2022). Environmental fate and toxicity of sunscreen-derived inorganic ultraviolet filters in aquatic environments: A review. Nanomaterials.

[42] Mueller N.C., Nowack B. (2008). Exposure modeling of engineered nanoparticles in the environment. Environ. Sci. Technol..

[43] Gottschalk F., Sonderer T., Scholz R.W., Nowack B. (2009). Modeled environmental concentrations of engineered nanomaterials (TiO_2_, ZnO, Ag, CNT, Fullerenes) for different regions. Environ. Sci. Technol..

[44] Dale A.L., Casman E.A., Lowry G.V., Lead J.R., Viparelli E., Baalousha M. (2015). Modeling nanomaterial environmental fate in aquatic systems. Environ. Sci. Technol..

[45] Tovar-Sanchez A., Sanchez-Quiles D., Basterretxea G., Benede J.L., Chisvert A., Salvador A., Moreno-Garrido I., Blasco J. (2013). Sunscreen products as emerging pollutants to coastal waters. PLoS ONE.

[46] Reed R.B., Martin D.P., Bednar A.J., Montano M.D., Westerhoff P., Ranville J.F. (2017). Multi-day diurnal measurements of Ti-containing nanoparticle and organic sunscreen chemical release during recreational use of a natural surface water. Environ. Sci. Nano..

[47] Gondikas A.P., Von Der Kammer F., Reed R.B., Wagner S., Ranville J.F., Hofmann T. (2014). Release of TiO_2_ nanoparticles from sunscreens into surface waters: A one-year survey at the old Danube recreational lake. Environ. Sci. Technol..

[48] Loosli F., Wang J., Rothenberg S., Bizimis M., Winkler C., Borovinskaya O., Flamigni L., Mohammed Baalousha M. (2019). Sewage spills are a major source of titanium dioxide engineered (nano)-particles into the environment. Environ Sci Nano..

[49] Mahlalela L.C., Ngila J.C., Dlamini L.N. (2017). Monitoring the fate and behavior of TiO_2_ nanoparticles: Simulated in a WWTP with industrial dye-stuff effluent according to OECD 303A. J. Environ. Sci. Health Part A.

[50] Zhang H., Sun Y., Tian A., Xue X.X., Wang L., Alquhali A., Bai X. (2013). Improved antibacterial activity and biocompatibility on vancomycin-loaded TiO_2_ nanotubes: In vivo and in vitro studies. Int. J. Nanomed..

[51] Vimbela G.V., Ngo S.M., Fraze C., Yang L., Stout D.A. (2017). Antibacterial properties and toxicity from metallic nanomaterials. Int. J. Nanomed..

[52] Rosli N.A., Teow Y.H., Mahmoudi E. (2021). Current approaches for the exploration of antimicrobial activities of nanoparticles. Sci. Technol. Adv. Mater..

[53] Liao C., Li Y., Tjong S.C. (2020). Visible-light active titanium dioxide nanomaterials with bactericidal properties. Nanomaterials.

[54] Parra-Ortiz E., Malmsten M. (2022). Photocatalytic nanoparticles—From membrane interactions to antimicrobial and antiviral effects. Adv. Colloid Interface Sci..

[55] Khater M.S., Kulkarni G.R., Khater S.S., Gholap H., Patil R. (2020). Study to elucidate effect of titanium dioxide nanoparticles on bacterial membrane potential and membrane permeability. Mater. Res. Express.

[56] Sohm B., Immel F., Bauda P., Pagnout C. (2015). Insight into the primary mode of action of TiO_2_ nanoparticles on *Escherichia coli* in the dark. Proteomics.

[57] Pagnout C., Jomini S., Dadhwal M., Caillet C., Thomas F., Bauda P. (2012). Role of electrostatic interactions in the toxicity of titanium dioxide nanoparticles toward *Escherichia coli*. Colloids Surf. B Biointerfaces.

[58] Leung Y.H., Xu X., Ma A.P., Liu F., Ng A.M., Shen Z., Gethings L.A., Guo M.Y., Djurišić A.B., Lee P.K. (2016). Toxicity of ZnO and TiO_2_ to *Escherichia coli* cells. Sci. Rep..

[59] Jiang W., Yang K., Vachet R.W., Xing B. (2010). Interaction between oxide nanoparticles and biomolecules of the bacterial cell envelope as examined by infrared spectroscopy. Langmuir.

[60] Wickham J.R., Rice C.V. (2008). Solid-state NMR studies of bacterial lipoteichoic acid adsorption on different surfaces. Solid State Nucl. Magn. Reson..

[61] Kumar A., Pandey A.K., Singh S.S., Shanker R., Dhawan A. (2011). Cellular uptake and mutagenic potential of metal oxide nanoparticles in bacterial cells. Chemosphere.

[62] Spirescu V.A., Chircov C., Grumezescu A.M., Vasile B.Ș., Andronescu E. (2021). Inorganic nanoparticles and composite films for antimicrobial therapies. Int. J. Mol. Sci..

[63] Akhtar S., Shahzad K., Mushtaq S., Ali I., Rafe M.H., Fazal-Ul-Karim S.M. (2019). Antibacterial and antiviral potential of colloidal Titanium dioxide (TiO_2_) nanoparticles suitable for biological applications. Mater. Res. Express.

[64] Cheigh C.I., Park M.H., Chung M.S., Shin J.K., Park Y.S. (2012). Comparison of intense pulsed light- and ultraviolet (UVC)-induced cell damage in *Listeria monocytogenes* and *Escherichia coli* O157:H7. Food Control.

[65] Van Grieken R., Marugán J., Pablos C., Furones L., López A. (2010). Comparison between the photocatalytic inactivation of Gram-positive *E. faecalis* and Gram-negative *E. coli* faecal contamination indicator microorganisms. Appl. Catal. B Environ..

[66] Kubacka A., Diez M.S., Rojo D., Bargiela R., Ciordia S., Zapico I., Albar J.P., Barbas C., Martins dos Santos V.A., Fernández-García M. (2014). Understanding the antimicrobial mechanism of TiO_2_-based nanocomposite films in a pathogenic bacterium. Sci. Rep..

[67] Kiwi J., Rtimi S. (2018). Mechanisms of the antibacterial effects of TiO_2_–FeOx under solar or visible light: Schottky barriers versus surface plasmon resonance. Coatings.

[68] Ekielski A. (2018). Interactions between food ingredients and nanocomponents used for composite packaging. Encyclopedia of Food Chemistry.

[69] Mba I.E., Nweze E.I. (2021). Nanoparticles as therapeutic options for treating multidrug-resistant bacteria: Research progress, challenges, and prospects. World J. Microbiol. Biotechnol..

[70] Ansari M.A., Albetran H.M., Alheshibri M.H., Timoumi A., Algarou N.A., Akhtar S., Slimani Y., Almessiere M.A., Alahmari F.S., Baykal A. (2020). Synthesis of electrospun TiO_2_ nanofibers and characterization of their antibacterial and antibiofilm potential against Gram-positive and Gram-negative bacteria. Antibiotics.

[71] Ahmed F.Y., Farghaly Aly U., Abd El-Baky R.M., Waly N.G.F.M. (2020). Comparative study of antibacterial effects of titanium dioxide nanoparticles alone and in combination with antibiotics on MDR *Pseudomonas aeruginosa* strains. Int. J. Nanomed..

[72] Lan W., Wang S., Zhang Z., Liang X., Liu X., Zhang J. (2021). Development of red apple pomace extract/chitosan-based films reinforced by TiO_2_ nanoparticles as a multifunctional packaging material. Int. J. Biol. Macromol..

[73] Roy A.S., Parveen A., Koppalkar A.R., Prasad M. (2010). Effect of nano-titanium dioxide with different antibiotics against methicillin-resistant *Staphylococcus aureus*. J. Biomater. Nanobiotechnol..

[74] Burhan Aldeen Abdulrahman N., Muhammad Nssaif Z. (2018). Antimicrobial activity of zinc oxide, titanium dioxide and silver nanoparticles against methicillin-resistant *Staphylococcus aureus* isolates. Tikrit J. Pure Sci..

[75] Nizami M.Z.I., Xu V.W., Yin I.X., Yu O.Y., Chu C.H. (2021). Metal and metal oxide nanoparticles in caries prevention: A review. Nanomaterials.

[76] Elsaka S.E., Hamouda I.M., Swain M.V. (2011). Titanium dioxide nanoparticles addition to a conventional glass-ionomer restorative: Influence on physical and antibacterial properties. J. Dent..

[77] Garcia-Contreras R., Scougall-Vilchis R.J., Contreras-Bulnes R., Sakagami H., Morales-Luckie R.A., Nakajima H. (2015). Mechanical, antibacterial and bond strength properties of nano-titanium-enriched glass ionomer cement. J. Appl. Oral Sci..

[78] Xu H., Liu B., Qi W., Xu M., Cui X., Liu J., Li Q. (2021). Combined impact of TiO_2_ nanoparticles and antibiotics on the activity and bacterial community of partial nitrification system. PLoS ONE.

[79] Sun D.S., Kau J.H., Huang H.H., Tseng Y.H., Wu W.S., Chang H.H. (2016). Antibacterial properties of visible-light-responsive carbon-containing titanium dioxide photocatalytic nanoparticles against Anthrax. Nanomaterials.

[80] Anvar A.A., Ahari H., Ataee M. (2021). Antimicrobial properties of food nanopackaging: A new focus on foodborne pathogens. Front. Microbiol..

[81] Othman S.H., Salam N.A., Zainal N., Basha R.K., Talib R.A. (2014). Antimicrobial activity of TiO_2_ nanoparticle-coated film for potential food packaging applications. Int. J. Photoenergy.

[82] Bodaghi H., Mostofi Y., Oromiehie A., Zamani Z., Ghanbarzadeh B., Costa C., Conte A., Del Nobile M.A. (2013). Evaluation of the photocatalytic antimicrobial effects of a TiO_2_ nanocomposite food packaging film by in vitro and in vivo tests. Food Sci. Technol..

[83] Nassiri R., MohammadiNafchi A. (2013). Antimicrobial and barrier properties of bovine gelatin films reinforced by nano TiO_2_. J. Chem. Health Risks.

[84] Gumiero M., Peressini D., Pizzariello A., Sensidoni A., Iacumin L., Comi G., Toniolo R. (2013). Effect of TiO_2_ photocatalytic activity in a HDPE-based food packaging on the structural and microbiological stability of a short-ripened cheese. Food Chem..

[85] Tang S., Wang Z., Li P., Li W., Li C., Wang Y., Chu P.K. (2018). Degradable and photocatalytic antibacterial Au-TiO_2_/sodium alginate nanocomposite films for active food packaging. Nanomaterials.

[86] Anaya-Esparza L.M., Montalvo-González E., González-Silva N., Méndez-Robles M.D., Romero-Toledo R., Yahia E.M., Pérez-Larios A. (2019). Synthesis and characterization of TiO_2_-ZnO-MgO mixed oxide and their antibacterial activity. Materials.

[87] Salahuddin N., Abdelwahab M., Gaber M., Elneanaey S. (2020). Synthesis and design of norfloxacin drug delivery system based on PLA/TiO_2_ nanocomposites: Antibacterial and antitumor activities. Mater. Sci. Eng. C Mater. Biol. Appl..

[88] Nandagopal S., Robin A., Soney C.G., Jayachandran V.P., Nandakumar K., Sabu T. (2016). Gentamicin loaded electrospun poly(ε-caprolactone)/TiO_2_ nanocomposite membranes with antibacterial property against Methicillin resistant *Staphylococus aureus* (MRSA). Polym. Plast. Technol. Eng..

[89] Ahmad A., Sabir A., Iqbal S.S., Felemban B., Riaz T., Bahadar A., Hossain N., Khan R.U., Inam F. (2022). Novel antibacterial polyurethane and cellulose acetate mixed matrix membrane modified with functionalized TiO_2_ nanoparticles for water treatment applications. Chemosphere.

[90] Franzin N.R.S., Sostena M.M.D.S., Santos A.D.D., Moura M.R., Camargo E.R., Hosida T.Y., Delbem A.C.B., Moraes J.C.S. (2022). Novel pulp capping material based on sodium trimetaphosphate: Synthesis, characterization, and antimicrobial properties. J. Appl. Oral Sci..

[91] Maurer-Jones M.A., Gunsolus I.L., Murphy C.J., Haynes C.L. (2013). Toxicity of engineered nanoparticles in the environment. Anal. Chem..

[92] Nel A.E., Mädler L., Velegol D., Xia T., Hoek E.M.V., Somasundaran P., Klaessig F., Castranova V., Thompson M. (2009). Understanding biophysicochemical interactions at the nano-bio interface. Nat. Mater..

[93] Niño-Martínez N., Salas Orozco M.F., Martínez-Castañón G.A., Torres Méndez F., Ruiz F. (2019). Molecular mechanisms of bacterial resistance to metal and metal oxide nanoparticles. Int. J. Mol. Sci..

[94] Planchon M., Ferrari R., Guyot F., Gélabert A., Menguy N., Chanéac C., Thill A., Benedetti M.F., Spalla O. (2013). Interaction between Escherichia coli and TiO_2_ nanoparticles in natural and artificial waters. Colloids Surf. B Biointerfaces.

[95] Ammendolia M.G., Iosi F., De Berardis B., Guccione G., Superti F., Conte M.P., Longhi C. (2014). *Listeria monocytogenes* behaviour in presence of non-UV-irradiated titanium dioxide nanoparticles. PLoS ONE.

[96] Zhang Q., Xia T., Zhang C. (2020). Chronic exposure to titanium dioxide nanoparticles induces commensal-to-pathogen transition in *Escherichia coli*. Environ. Sci. Technol..

[97] Zhang J., Wang X., Suo X., Liu X., Liu B., Yuan M., Wang G., Liang C., Shi H. (2019). Cellular response of *Escherichia coli* to photocatalysis: Flagellar assembly variation and beyond. ACS Nano.

[98] Zhu J., Wang J., Chen Y.P., Qing T., Zhang P., Feng B. (2022). Quantitative proteomics and phosphoproteomics elucidate the molecular mechanism of nanostructured TiO_2_-stimulated biofilm formation. J. Hazard Mater..

[99] Mathur A., Kumari J., Parashar A.T.L., Chandrasekaran N., Mukherjee A. (2015). Decreased phototoxic effects of TiO_2_ nanoparticles in consortium of bacterial isolates from domestic waste water. PLoS ONE.

[100] Zhu N., Wang S., Tang C., Duan P., Yao L., Tang J., Wong P.K., An T., Dionysiou D.D., Wu Y. (2019). Protection mechanisms of periphytic biofilm to photocatalytic nanoparticle exposure. Environ. Sci. Technol..

[101] Farkas J., Peter H., Ciesielski T.M., Thomas K.V., Sommaruga R., Salvenmoser W., Weyhenmeyer G.A., Tranvik L.J., Jenssen B.M. (2015). Impact of TiO_2_ nanoparticles on freshwater bacteria from three Swedish lakes. Sci. Total Environ..

[102] Zhai Y., Chen L., Liu G., Song L., Arenas-Lago D., Kong L., Peijnenburg W., Vijver M.G. (2021). Compositional and functional responses of bacterial community to titanium dioxide nanoparticles varied with soil heterogeneity and exposure duration. Sci. Total Environ..

[103] Wu J., Zhan M., Chang Y., Su Q., Yu R. (2018). Adaption and recovery of Nitrosomonas europaea to chronic TiO_2_ nanoparticle exposure. Water Res..

[104] Maurer-Jones M.A., Gunsolus I.L., Meyer B.M., Christenson C.J., Haynes C.L. (2013). Impact of TiO_2_ nanoparticles on growth, biofilm formation, and flavin secretion in *Shewanella oneidensis*. Anal. Chem..

[105] Ahmed F.Y., Aly U.F., Abd El-Baky R.M., Waly N.G.F.M. (2021). Effect of titanium dioxide nanoparticles on the expression of efflux pump and quorum-sensing genes in MDR *Pseudomonas aeruginosa* isolates. Antibiotics.

[106] Pagnout C., Razafitianamaharavo A., Sohm B., Caillet C., Beaussart A., Delatour E., Bihannic I., Offroy M., Duval J.F.L. (2021). Osmotic stress and vesiculation as key mechanisms controlling bacterial sensitivity and resistance to TiO_2_ nanoparticles. Commun. Biol..

[107] Eymard-Vernain E., Luche S., Rabilloud T., Lelong C. (2018). Impact of nanoparticles on the *Bacillus subtilis* (3610) competence. Sci. Rep..

[108] Qiu Z., Shen Z., Qian D., Jin M., Yang D., Wang J., Zhang B., Yang Z., Chen Z., Wang X. (2015). Effects of nano-TiO_2_ on antibiotic resistance transfer mediated by RP4 plasmid. Nanotoxicology.

[109] Xiao X., Ma X.L., Han X., Wu L.J., Liu C., Yu H.Q. (2021). TiO2 photoexcitation promoted horizontal transfer of resistance genes mediated by phage transduction. Sci. Total. Environ..

[110] Barnowska-Wójcik E. (2021). Factors conditioning the potential effects TiO_2_ NPs exposure on human microbiota: A mini-review. Biol. Trace Elem. Res..

[111] Taylor A.A., Marcus I.M., Guysi R.L., Walker S.L. (2015). Metal oxide nanoparticles induce minimal phenotypic changes in a model colon gut microbiota. Environ. Eng. Sci..

[112] Pinget G., Tan J., Janac B., Kaakoush N.O., Angelatos A.S., O’Sullivan J., Koay Y.C., Sierro F., Davis J., Divakarla S.K. (2019). Corrigendum: Impact of the food additive titanium dioxide (E171) on gut microbiota-host interaction. Front. Nutr..

[113] Waller T., Chen C., Walker S.L. (2017). Food and industrial grade titanium dioxide impacts gut microbiota. Environ. Eng. Sci..

[114] Agans R.T., Gordon A., Hussain S., Paliy O. (2019). Titanium dioxide nanoparticles elicit lower direct inhibitory effect on human gut microbiota than silver nanoparticles. Toxicol. Sci..

[115] Planchon M., LeÂger T., Spalla O., Huber G., Ferrari R. (2017). Metabolomic and proteomic investigations of impacts of titanium dioxide nanoparticles on *Escherichia coli*. PLoS ONE.

[116] Dudefoi W., Moniz K., Allen-Vercoe E., Ropers M.H., Walker V.K. (2017). Impact of food grade and nano-TiO_2_ particles on a human intestinal community. Food. Chem. Toxicol..

[117] Yang F., Liu S.L., Xu Y., Walker S.G., Cho W., Mironava T., Rafailovich M. (2021). The impact of TiO_2_ nanoparticle exposure on transmembrane cholesterol transport and enhanced bacterial infectivity in HeLa cells. Acta Biomater..

[118] Ammendolia M.G., De Berardis B., Maurizi L., Longhi C. (2020). Exposure to TiO_2_ nanoparticles increases *Listeria monocytogenes* infection of intestinal epithelial cells. Nanomaterials.

[119] Nabavizadeh M., Abbaszadegan A., Gholami A., Kadkhoda Z., Mirhadi H., Ghasemi Y., Safari A., Hemmateenejad B., Dorostkar S., Sharghi H. (2017). Antibiofilm efficacy of positively charged imidazolium-based silver nanoparticles in *Enterococcus faecalis* using quantitative real-time PCR. Jundishapur J. Microbiol..

[120] Abbaszadegan A., Ghahramani Y., Gholami A., Hemmateenejad B., Dorostkar S., Nabavizadeh M., Sharghi H. (2015). The effect of charge at the surface of silver nanoparticles on antimicrobial activity against gram-positive and gram-negative bacteria: A preliminary study. J. Nanomater..

[121] Di Bonaventura G., Piccolomini R., Paludi D., D’Orio V., Vergara A., Conter M., Ianieri A. (2008). Influence of temperature on biofilm formation by *Listeria monocytogenes* on various food-contact surfaces: Relationship with motility and cell surface hydrophobicity. J. Appl. Microbiol..

[122] Raza S., Matuła K., Karoń S., Paczesny J. (2021). Resistance and adaptation of bacteria to non-antibiotic antibacterial agents: Physical stressors, nanoparticles, and bacteriophages. Antibiotics.

[123] Fasnacht M., Polacek N. (2021). Oxidative stress in bacteria and the central dogma of molecular biology. Front. Mol. Biosci..

[124] Yin H., Li G., Chen X., Wang W., Wong P.K., Zhao H., An T. (2020). Accelerated evolution of bacterial antibiotic resistance through early emerged stress responses driven by photocatalytic oxidation. Appl. Catal. B-Environ..

[125] Gunawan C., Lim M., Marquis C.P., Amal R. (2014). Nanoparticle–protein corona complexes govern the biological fates and functions of nanoparticles. J. Mater. Chem. B.

